# Quantification of pulmonary disease activity in sarcoidosis measured with ^18^F-FDG PET/CT: SUVmax versus total lung glycolysis

**DOI:** 10.1186/s13550-019-0505-x

**Published:** 2019-06-13

**Authors:** Milou C. Schimmelpennink, Adriane D. M. Vorselaars, Marcel Veltkamp, Ruth G. M. Keijsers

**Affiliations:** 1 Interstitial Lung Diseases Center of Excellence, Department of Pulmonology, St Antonius Hospital, Koekoekslaan 1, 3435 CM Nieuwegein, the Netherlands; 2 Division of Heart and Lungs, University Medical Center, Utrecht, the Netherlands; 3 Department of Nuclear Medicine, St Antonius Hospital, Nieuwegein, the Netherlands

**Keywords:** ^18^F-FDG PET/CT, Sarcoidosis, Total lung glycolysis (TLuG), Standardized uptake value (SUVmax)

## Abstract

**Background:**

^18^F-FDG PET/CT has proven to be a reliable tool for therapy monitoring in sarcoidosis. Previous PET studies investigated the SUVmax as a marker for disease activity. Total lung glycolysis (TLuG) is a new tool, quantifying the glycolysis of the entire lung. Since SUVmax represents the maximum activity in only one pixel, we hypothesize that TLuG is a more accurate marker for active pulmonary disease and predictor of response than SUVmax.

**Methods:**

In this retrospective cohort study, 27 patients started on infliximab for refractory pulmonary sarcoidosis. Patients received infliximab intravenously monthly at a dose of 5 mg/kg. We performed a lung function test and an ^18^F-FDG PET/CT before initiation of infliximab and after 6 months of treatment. SUVmax and TLuG were determined in the pre- and post-scan. Change in lung function was correlated with the change in SUVmax and TLuG and was correlated to the initial SUVmax and TLuG to evaluate the predictive value of the initial metabolic activity.

**Results:**

ΔSUVmax significantly correlated with ΔFVC (*r* = − 0.497, *p* = 0.008) and with ΔFEV1 (*r* = − 0.467, *p* = 0.014). Furthermore, ΔTLuG significantly correlated with ΔFVC (*r* = − 0.430, *p* = 0.025), ΔFEV1 (*r* = − 0.532, *p* = 0.004) and ΔDLCOc (*r* = − 0.423, *p* = 0.039). Change in SUVmax and TLuG significantly correlated (*r* = 0.735, *p* < 0.001). Initial SUVmax significantly correlated with the change in FVC and DLCOc. In addition, initial TLuG significantly correlated with the change in FEV1 and DLCOc.

A SUVmax > 7.5 at initiation of infliximab was predictive for 5% response in FVC, whereas SUVmax > 9.2 was predictive for 5% response in DLCOc. In addition, high TLuG > 4100 at initiation of infliximab was predictive for 5% response in FVC and FEV1 and TLuG > 4500 was predictive for response in DLCOc.

**Conclusion:**

SUVmax and TLuG are equal in determining the response to infliximab in pulmonary sarcoidosis patients. Furthermore, SUVmax and TLuG at initiation of infliximab can predict change in lung function after treatment. Since TLuG is a more time-consuming tool, we recommend to use SUVmax of the lung parenchyma for response monitoring in pulmonary sarcoidosis.

## Background

Sarcoidosis is a granulomatous multi-systemic disease with both a heterogeneous presentation and clinical course [1]. Several biomarkers are determined in the standard diagnostic work-up and follow-up of patients with sarcoidosis, like serum angiotensin-converting enzyme (ACE) and soluble interleukin 2 receptor (sIL-2R) in serum, as well as lymphocytes and CD4^+^/CD8^+^ ratio in bronchoalveolar lavage [2–4].

^18^F-FDG PET/CT has proven to be a reliable biomarker to measure disease activity in sarcoidosis [5, 6] and to detect occult sarcoidosis lesions [7, 8].

Maximum standardized uptake value (SUVmax) is the most commonly used semi-quantitative value of ^18^F-FDG PT/CT in sarcoidosis. In clinical oncology, total lesion glycolysis can additionally be used to quantify activity on ^18^F-FDG PET/CT. Total lesion glycolysis is measured as the product of the mean standardized uptake value (SUVmean) and the metabolic volume of the lesion. Total lesion glycolysis is used in the standard follow-up in patients with malignancies for response rating after treatment [9]. Furthermore, total lesion glycolysis has proven to be a better prognostic marker than SUVmax in patients with malignancies [10]. As SUVmax is only derived from activity in one pixel, it is insufficient to objectify the global inflammation of the lungs. Total lung glycolysis is a new tool that is a derivative of the total lesion glycolysis focused on the lungs.

To our knowledge, no studies have been performed investigating the response rate of sarcoidosis patients to infliximab using the semi-quantitative total glycolysis of the lung (TLuG). We hypothesize that determining the amount of inflammatory activity in pulmonary sarcoidosis will be more accurate by using TLuG than by SUVmax. The aim of our study is to compare the prognostic value of SUVmax and TLuG regarding the change in lung function in pulmonary sarcoidosis patients treated with infliximab.

## Methods

### Study population

This study is a retrospective cohort study consisting of 27 patients with refractory pulmonary sarcoidosis indicated for infliximab treatment. All consecutive patients started infliximab therapy between July 2010 and September 2015. Sarcoidosis was defined as refractory when organ damage persisted while receiving second-line immunosuppressive treatment or when second-line therapy had to be discontinued due to toxicity.

All patients received infliximab at a dose of 5 mg/kg intravenously at week 0, week 2 and thereafter every 4 weeks. Lung function and ^18^F-FDG PET/CT were routinely performed before and after the induction phase of 26 weeks. Sarcoidosis was diagnosed according to the guidelines of ATS/ERS/WASOG statement [11]. The following data were extracted from patient records: gender, race, smoking history, organ involvement and Scadding stage. The study was approved by the investigational review board of St Antonius Hospital Nieuwegein (registration number LTME/Z-12.033 and acronym ORATS).

### Lung function

Lung function was performed before and 6 months after induction of infliximab. Lung function tests were performed using Master Screen Body (Jaeger ms-pft analyse unit, Würzberg, Germany). Forced vital capacity (FVC), forced expiratory volume in 1 s (FEV1) and diffusing capacity of the lung for carbon monoxide corrected for haemoglobin (DLCOc) were expressed as percentages of predicted. Furthermore, the change of the pulmonary function parameters after 26 weeks of infliximab treatment was measured and expressed as ΔFVC, ΔFEV1 and ΔDLCOc.

### ^18^F-FDG PET/CT

^18^F-FDG PET/CT was performed in accordance with the joint guideline of the Society of Nuclear Medicine and European Association of Nuclear Medicine [12]. FDG PET/CT was performed with a Philips Gemini Time of Flight PET/CT scanner (Philips Medical Systems, Best, the Netherlands). The Department of Nuclear Medicine of the St Antonius Hospital is an EARL accredited PET/CT centre. Low-dose CT was used for attenuation correction and optimizing image interpretation. Reconstruction of the PET images is performed in accordance with the 3D–row action maximum likelihood algorithm protocol (RAMLA), applying four iterations with a 144 × 144 matrix.

A quadratic FDG dosage regimen was used based on the patient’s body weight with a minimum of 37 MBq and a maximum of 400 MBq. Emission scan was performed from the subinguinal region to the head.

The SUVmax was determined by two observers (RK and MS). The SUVmax was calculated in the lung parenchyma as described before [13]. Region of interest (ROI) was drawn over the visually affected part of the organ to measure the SUVmax. ROI was drawn at the same lesion/area at baseline and follow-up scan after infliximab. ROI drawing was performed using the automatic ROI drawing tool in the Hermes Diagnostics programme (Hermes Medical Solutions, Stockholm, Sweden).

TLuG, the total lung glycolysis, is a derivative of the total lesion glycolysis (TLG). In contrast with TLG, focusing on a lesion, TLuG is focused on an organ, i.e. the lung.

**Fig. 1:**
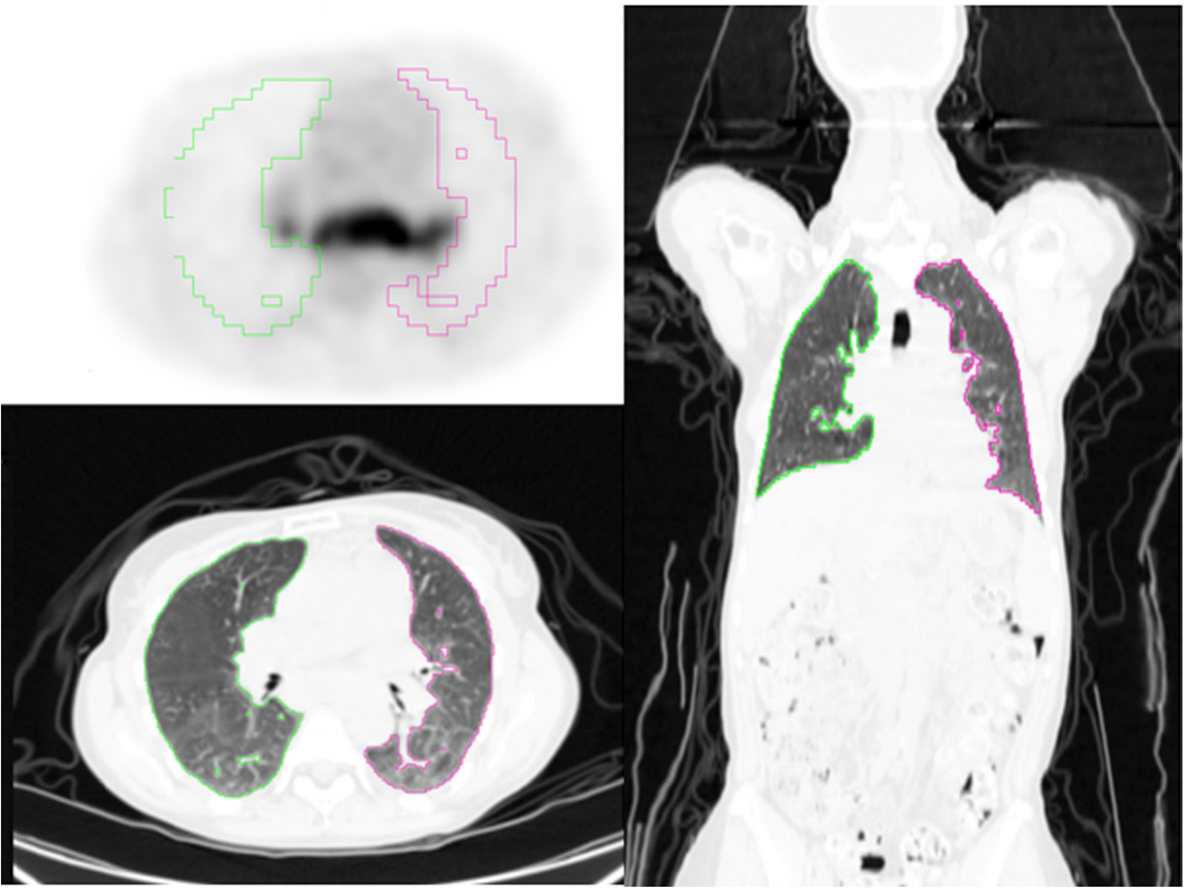
Example of VOI of total lung glycolysis. TLuG is the cumulative metabolic activity in the total lung parenchyma. Additionally, SUVmax and SUVmean are determined in the VOI

The TLuG provides information regarding the cumulative metabolic activity in the lung parenchyma, as described previously in the paper of Adams et al. [14]. TLuG was determined by two nuclear medicine physicians (RK and HA). The lung parenchyma is therefore our volume of interest (VOI) (Fig. 1). This VOI was determined semi-automatically by CT based on Hounsfield units (HU) in accordance with Adams et al. [14]. VOI was measured by using a lung segmentation programme provided by Hermes Medical Solutions (Stockholm, Sweden). This CT-based VOI served as a demarcated volume in PET in which the total metabolic activity was measured, expressed as TLuG, SUVmean and SUVmax.

### Statistics

All analyses were performed using IBM SPSS Statistics 24. Continuous variables were expressed as mean ± standard deviation. Changes between baseline outcomes and outcomes after treatment with infliximab were analysed with the two-tailed paired *t* test. Correlation between the change in lung function (ΔFVC, ΔFEV1, ΔDLCOc) and change in SUVmax (ΔSUVmax) and TLuG (ΔTLuG) was measured by the Pearson correlation coefficient. Pearson correlation coefficient (expressed as *r*) of 0.9–1.00 was considered as a very high correlation, 0.7–0.9 high correlation, 0.5–0.7 moderate, 0.3–0.5 low and 0.00–0.30 negligible [15]. The inter-observer variability for TLuG was measured with the intraclass correlation coefficient.

The optimal cut-off point of SUVmax and TLuG to predict 5% response in lung function (5% FVC, FEV1, DLCOc % of predicted) was found by maximizing the area under the curve (AUC) of the receiver operating characteristic (ROC) curve. Cut-off values of SUVmax and TLuG were selected with the maximum value of Youden index (Youden index = sensitivity + specificity − 1). Subsequently, we rounded these values to a clinically useful value.

## Results

### Study population

Characteristics of all patients are presented in Table 1. ^18^F-FDG PET/CT and lung function at baseline and after 6 months of infliximab treatment were available from all 27 patients with pulmonary refractory sarcoidosis, with the exception of DLCOc in 3 patients.

**Table 1:** Characteristics of patients, *n* = 27

		Patient characteristics
Age		48.1 ± 10.0 years
Gender (male)		17 (63.0%)
Caucasian		24 (88.9%)
Smoking history	Current	4 (14.8%)
	Former	13 (48.1%)
	Non-smoker	10 (37.0%)
Scadding stages at initiation of infliximab	I	1 (3.7%)^a^
	II	5 (18.5%)
	III	4 (14.8%)
	IV	17 (63%)

^a^Treatment indication for infliximab in this patient was severe obstructive pulmonary function caused by endobronchial stenosing

Lung function parameters and both semi-quantitative metabolic values on ^18^F-FDG PET/CT, SUVmax and TLuG, at baseline and after 26 weeks of infliximab therapy, are shown in Table 2.

**Table 2:** Pulmonary function, SUVmax and TLuG at baseline and after 26 weeks infliximab treatment, *n* = 27; mean ± SD

	Baseline	After 26 weeks infliximab	Change	*p* value
FVC (% predicted)	75.1 ± 18.4	79.7 ± 19.9	+ 4.6 ± 8.4	0.009
FEV1 (% predicted)	58.6 ± 17.9	63.6 ± 20.5	+ 5.1 ± 6.8	0.001
DLCOc (% predicted)^a^	55.5 ± 17.9	57.9 ± 16.9	+ 2.4 ± 6.8	0.100
SUVmax	8.2 ± 4.7	3.1 ± 2.9	− 5.1 ± 5.1	< 0.001
TLuG	5395 ± 3216	2641 ± 952	− 2755 ± 3064	< 0.001

*FVC* forced vital capacity, *FEV1* forced expiratory volume in 1 s, *DLCOc* diffusing capacity of the lung for carbon monoxide corrected for haemoglobin, *SUVmax* maximum standardized uptake value, *TLuG* total lung glycolysis

^a^Three missing values

After 6 months of treatment with infliximab, FVC and FEV1 significantly increased, + 4.6% and + 5.1% predicted respectively (*p* = 0.009 and *p* = 0.001). Furthermore, the DLCOc increased with 2.4%; however, this did not reach significance.

Both SUVmax and TLuG in the lung parenchyma reduced significantly after therapy with infliximab. SUVmax decreased with 59% from 8.1 ± 4.9 to 3.3 ± 2.9 (*p* < 0.001). TLuG decreased with 51% from 5395 to 2641 (*p* < 0.001). There was a very high inter-observer agreement for TLuG measurements and SUVmax measurements, with an intraclass correlation coefficient of 0.963 (CI interval 0.917–0.983) (*p* < 0.001) for TLuG and an intraclass coefficient of 0.956 (CI interval 0.906–0.980) for SUVmax.

### Correlation between quantification of inflammatory activity measured by ^18^F-FDG PET and lung function

Correlations between the change in SUVmax and TLuG and the change in lung function parameters are shown in Table 3.

**Table 3:** Correlation of the change in SUVmax and TLuG with the change in lung function parameters, *n* = 27

Correlation tested *R* (*p* value)	ΔSUVmax	ΔTLuG
ΔFVC	− 0.497 (*p* = 0.008)	− 0.430 (*p* = 0.025)
ΔFEV1	− 0.467 (*p* = 0.014)	− 0.532 (*p* = 0.004)
ΔDLCOc	− 0.391 (*p* = 0.059)	− 0.423 (*p* = 0.039)

Δ change before and after infliximab therapy, *FVC* forced vital capacity, *FEV1* forced expiratory volume in 1 s, *DLCOc* diffusing capacity of the lung for carbon monoxide corrected for haemoglobin, *SUVmax* maximum standardized uptake value, *TLuG* total lung glycolysis

**Fig. 2:**
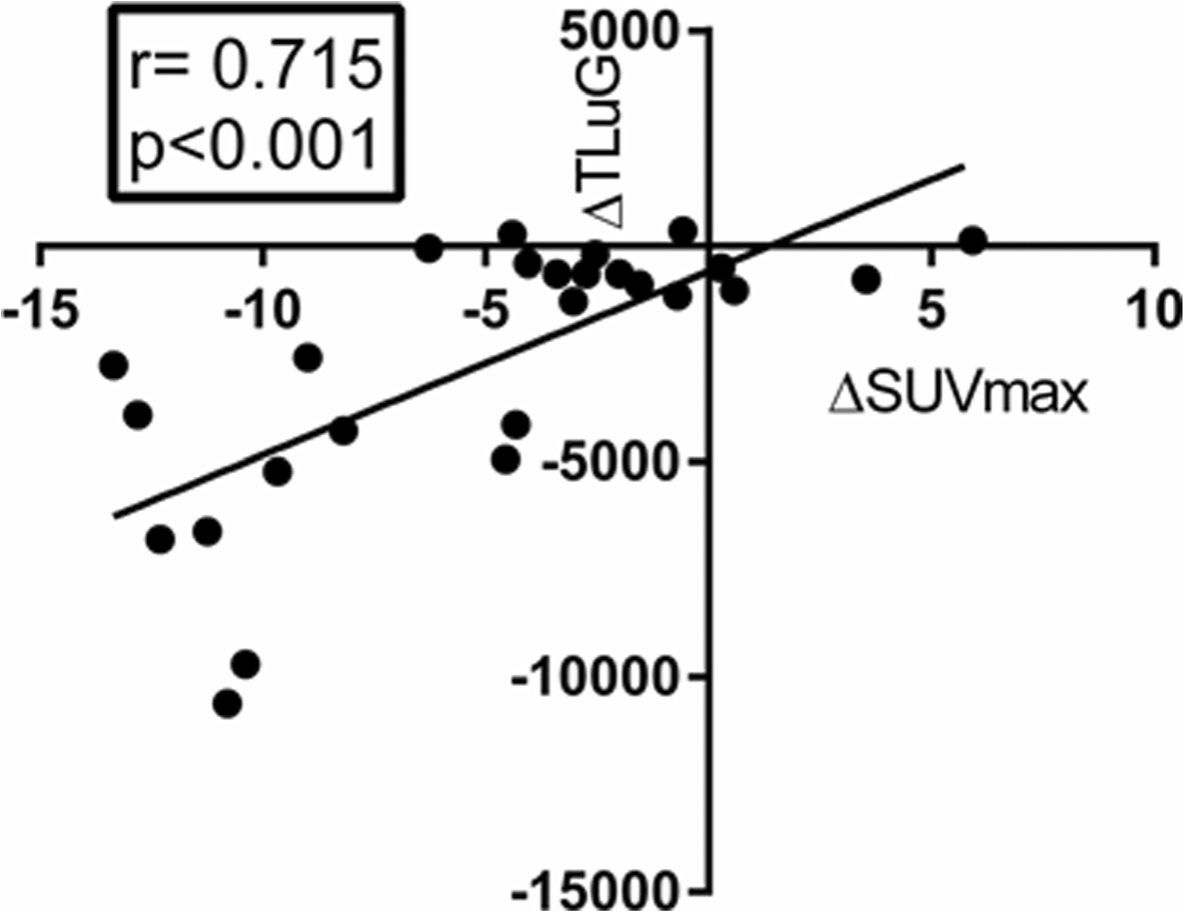
Correlation of the change in SUVmax and TLuG

Change in SUVmax and TLuG during infliximab therapy correlated significantly (*r* = 0.735, *p* < 0.001) (Fig. 2).

A significant correlation was found between ΔSUVmax and ΔFVC during therapy (*r* = − 0.497, *p* = 0.008) and between ΔSUVmax and ΔFEV1 (*r* = − 0.467, *p* = 0.014). No correlation was found between ΔSUVmax and ΔDLCOc.

ΔTLuG and ΔFVC as well as ΔTLuG and ΔDLCOc showed a correlation (*r* = − 0.430, *p* = 0.025; *r* = − 0.423, *p* = 0.039 respectively). In addition, a significant correlation was also found between ΔTLuG and ΔFEV1 (*r* = − 0.532, *p* = 0.004).

### Prognostic value of baseline TLuG and SUVmax

Correlations between the baseline SUVmax and TLuG and the change of lung function parameters are shown in Table 4.

**Table 4:** Correlation of baseline SUVmax and TLuG with change in lung function parameters, *n* = 27

Correlation tested *R* (*p* value)	Baseline SUVmax	Baseline TLuG
ΔFVC	0.460 (*p* = 0.016)	0.323 (*p* = 0.100)
ΔFEV1	0.344 (*p* = 0.079)	0.430 (*p* = 0.025)
ΔDLCOc^a^	0.513 (*p* = 0.010)	0.453 (*p* = 0.026)

Δ change before and after infliximab therapy, *FVC* forced vital capacity, *FEV1* forced expiratory volume in 1 s, *DLCOc* diffusing capacity of the lung for carbon monoxide corrected for haemoglobin, *SUVmax* maximum standardized uptake value, *TLuG* total lung glycolysis

^a^Three missing values

A significant correlation was found between baseline SUVmax and ΔFVC (*r* = 0.460, *p* = 0.016) as well as baseline SUVmax and ΔDLCOc (*r* = 0.513, *p* = 0.010). No correlation was found between baseline SUVmax and ΔFEV1.

No correlation was found between baseline TLuG and ΔFVC, although a significant correlation was found between baseline TLuG and ΔFEV1 (*r* = 0.430, *p* = 0.025) and baseline TLuG and ΔDLCOc (*r* = 0.453, *p* = 0.026).

ROC curves were determined in order to select the best cut-off value of SUVmax and TLuG to predict lung functional response of 5% predicted FVC, FEV1 and DLCOc.

The optimal cut-off value of SUVmax to predict a 5% response in FVC was 7.5, with an AUC of 0.773 (95% CI 0.594–0.951, *p* = 0.018). And the optimal cut-off value of SUVmax to predict 5% response in DLCOc was 9.2, with an AUC of 0.763 (95% CI 0.557–0.698, *p* = 0.034).

The optimal cut-off value of TLuG to predict response of 5% FVC and FEV1 was 4100, with an AUC of 0.739 (95% CI 0.540–0.937) and 0.739 (95% CI 0.544–0.934) (*p* = 0.038 and *p* = 0.035). Furthermore, the optimal cut-off value of TLuG to predict response of 5% DLCOc was 4500, with an AUC of 0.744 (95% CI 0.542–0.947, *p* = 0.049).

### Discordant response

**Fig. 3:**
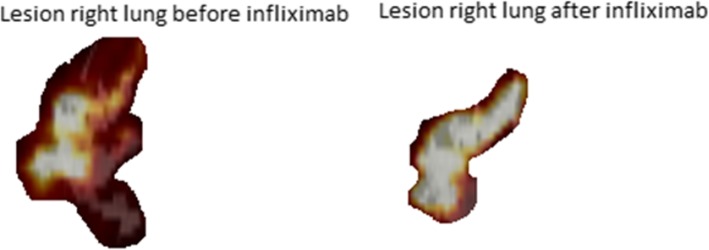
Patient A. Pre- and post-^18^F-FDG PET/CT, lesion in the right lung with a significant decrease in TLuG and a significant increase in DLCOc (% predicted), while SUVmax increases. Patient A showed persistent extensive parenchymal and endobronchial involvement with an impaired diffusion capacity despite corticosteroid treatment. Infliximab was initiated, and after 6 months of treatment, diffusion capacity increased with 9.8%. Furthermore, TLuG decreased with 11.8%. However, in contrast to TLuG, the SUVmax increased with + 32.4%

A discordant response was shown in only four patients (Table 5). One patient showed a decrease in SUVmax, whereas the TLuG increased. And in three patients, the SUVmax increased, whereas a decrease in TLuG was shown. Figure 3 shows an example of a patient with a discordant response.

**Table 5:** Discordant response in SUVmax and TLuG in four patients

	SUVmax			TLuG			ΔLung function		
	Pre	Post	Δ (%)	Pre	Post	Δ (%)	ΔFVC (%)	ΔFEV1 (%)	ΔDLCoc (%)
Pt A	11.2	14.7	+ 32.4	6606	5826	− 11.8	− 0.7	− 5.1	+ 9.8
Pt C	2.1	1.6	− 23.4	1827	2178	+ 19.2	− 4.1	− 5.3	0.0
Pt D	6.2	6.8	+ 9.7	4616	3596	− 22.1	− 10.7	− 8.1	− 4.8
Pt E	0.6	0.9	+ 50	2999	2555	− 16.8	− 16.7	− 2.2	+ 4.1

Δ change before and after infliximab therapy, *FVC* forced vital capacity, *FEV1* forced expiratory volume in 1 s, *DLCOc* diffusing capacity of the lung for carbon monoxide corrected for haemoglobin, *SUVmax* maximum standardized uptake value, *TLuG* total lung glycolysis

## Discussion

In recent years, multiple studies in sarcoidosis patients have described the use of SUVmax to quantify the sarcoidosis activity on a ^18^F-FDG PET as a biomarker [16, 17]. TLuG was determined by two observers and showed a very high inter-observer agreement, which implicates that TLuG measurements are reliable. This study demonstrates that change in SUVmax and change in TLuG correlate with change in lung function. No significant difference in correlation coefficient was found. This indicates that both semi-quantitative values, SUVmax and TLuG, can be used to monitor respiratory response to third-line treatment in sarcoidosis with infliximab.

A discordant response in TLuG and SUVmax was seen in only 4 of the 27 patients, whereas we hypothesized that TLuG would be a more sensitive marker for response measuring than SUVmax.

The discordant response in those 4 patients could be due to different patterns of parenchymal involvement in pulmonary sarcoidosis. For example, in patients with diffuse alveolar sarcoidosis, TLuG might be a better parameter, whereas in patients with one or more dense parenchymal infiltrates with high metabolic activity, SUVmax could be a better reflection of disease activity. Moreover, a discordant response can be found in patients with extensive involvement of the lung parenchyma. When the extent of the lesion decreases after therapy, the TLuG decreases while the maximum intensity of ^18^F-FDG uptake in one pixel, i.e. SUVmax, may remain unchanged.

In the second part of our study, we evaluated the prognostic value of SUVmax and TLuG at baseline to predict the change in lung function during infliximab treatment. Earlier studies have already focused on the prognostic value of ^18^F-FDG PET/CT in sarcoidosis. Adams et al. showed that SUVmax is a predictor for future deterioration of the diffusing capacity of the lung [18].

This study has a few limitations. First, the small sample size of the cohort reduces the power of the study. Also, due to the retrospective design of this study, there were a few missing data. In addition, long-term follow-up data is only available from a part of the patients in the study cohort; therefore, it remains unknown if TLuG is predictive for disease relapse, as previously shown for SUVmax.

## Conclusions

In conclusion, SUVmax and TLuG are both adequate markers to quantify the metabolic response to infliximab in pulmonary sarcoidosis patients. Both SUVmax and TLuG correlate with lung function change during therapy. In addition, SUVmax and TLuG can predict lung functional improvement to be achieved by infliximab.

In contrast with our hypothesis, TLuG was not superior compared to SUVmax. Based on these data, we recommend to use SUVmax over TLuG in evaluating sarcoidosis activity in the lung parenchyma.

## Abbreviations

^18^F-FDG PET/CT: (^18^F) fluoro-2-deoxy-d-glucose-positron emission tomography/computed tomography
ACE: Angiotensin-converting enzyme
AUC: Area under the curve
CD4^+^/CD8^+^ ratio: Ratio of T helper cells (with the surface marker CD4) to cytotoxic T cells (with the surface marker CD8)
DLCOc: Diffusing capacity of the lung for carbon monoxide corrected for haemoglobin
FEV1: Forced expiratory volume in 1 s
FVC: Forced vital capacity
ROC: Receiver operating characteristic
sIL-2R: Soluble interleukin 2 receptor
SUVmax: Maximum standardized uptake value
SUVmean: Mean standardized uptake value
TLG: Total lesion glycolysis
TLUG: Total lung glycolysis
